# *In silico* design and testing of a multi-epitope camel mastitis vaccine candidate for development

**DOI:** 10.3389/fcimb.2026.1753101

**Published:** 2026-05-29

**Authors:** Edwin Murungi, Nathan Langat, Ednah Masila, Ruth Onywera, Irene Ogali, Nimmo Gicheru, Hezron Wesonga, Monicah Maichomo

**Affiliations:** 1 Department of Medical Biochemistry, Kisii University, Kisii, Kenya; 2 Veterinary Sciences Research Institute, Kenya Agricultural and Livestock Research Organization, Muguga, Kenya; 3 Animals and Human Health Department, International Livestock Research Institute, Nairobi, Kenya

**Keywords:** camel mastitis, epitope, Staphylococcus aureus, Streptococcus agalactiae, vaccine

## Abstract

**Introduction:**

Camel mastitis caused by a diverse array of pathogenic bacteria including *Staphylococcus aureus* and *Streptococcus agalactiae* is responsible for enormous socioeconomic losses in the arid and semi-arid regions of Africa where camels are an invaluable source of livelihood and nutrition. Although *S. aureus* and *S. agalactiae* are environmental contaminants, the latter is an infectious zoonotic agent. The development of a safe, efficacious vaccine for camel mastitis is a compelling priority particularly against the backdrop of climate change and antimicrobial resistance.

**Methods:**

We have sequenced and assembled the genomes of local strains of *S. aureus*, *S. agalactiae*, *L. lactis*, *E. faecium* and *E. gallinarum* isolated from milk samples of pastoralists’ camel herds in Kenya, analysed the subtracted proteomes for antigenic proteins using VaxiJen and ascertained the allergenicity, subcellular localisation and the number of transmembrane helices of the putative candidates using AllerTOP, PSORTb and DeepTMHMM. Furthermore, B-cell and T-cell epitopes uncovered in the antigenic protein sequences using Bepi-Pred-2.0, NetCTL 1.2 and NetMHCII 2.3 were used to design a potential vaccine candidate that exhibited adequate immunogenicity, stability and flexibility without toxicity and allergenicity.

**Results:**

We have designed and computationally evaluated a multi-epitope camel mastitis vaccine candidate from 20 antigenic S. aureus and S. agalactiae proteins. The candidate exhibits desirable immunogenicity, is non-allergenic and non-toxic, and warrants further experimental investigations.

**Discussion:**

Collectively, we have designed and computationally evaluated a potentially viable camel mastitis vaccine candidate.

## 1 Introduction

Dromedary camels (*Camelus dromedarius*) are resilient, multi-purpose animals well adapted for survival in harsh environments characterized by scarcity of water and pasture (Darwish, 2023; Seligsohn et al., 2020). Camels are a vital source of income, milk and meat for pastoralists in Arid and Semi-Arid Lands (ASALs) of Africa (Guliye et al., 2007; Volpato and Howard, 2014) with camel milk being an important alternative source of protein and a particularly appropriate functional food for infants and geriatrics due to its high nutrients content in these regions (Alhaj and Al Kanhal, 2010; Rahmeh et al., 2022). Moreover, camel milk has been reported as a potential remedy for jaundice, tuberculosis, diabetes, asthma and leishmaniasis (Swelum et al., 2021).

Mastitis, an intramammary infection characterized by physical, chemical and bacteriological alterations in milk, and pathological changes in the glandular tissue (El Tigani-Asil et al., 2020) causes enormous agricultural losses due to diminished milk production, poor quality milk and microbial contamination of milk (Dzayee et al., 2022). Besides hampering animal productivity and significantly reducing farm income, the disease also adversely affects human health (Aqib et al., 2022; Seligsohn et al., 2020). The infection may be clinical (acute or chronic) or sub-clinical. Broadly, clinical mastitis is characterized by hardening, swelling and pain of the mammary gland coupled with discoloration and clotting of milk. Specifically, in acute mastitis, the mammary secretions are watery, yellowish and blood-tinged while fibrosis and keratinization of the udder tissue is prevalent in chronic mastitis (El Tigani-Asil et al., 2020). On the other hand, in sub-clinical mastitis, inflammation without overt symptoms prevails (Aqib et al., 2022). Clinical and sub-clinical camel mastitis diminish the yield, alter the properties, and impair the preservation and processing of milk (Kashongwe et al., 2017).

Bacterial infections are the commonest cause of camel mastitis (Seifu and Tafesse, 2010). Although an array of bacterial species including *Streptococcus* sp.*, Staphylococci* sp.*, Salmonella* sp.*, Enterococcus sp, Escherichia* sp.*, Mycoplasma* sp.*, Corynebacterium* sp.*, Pseudomonas* sp., and *Campylobacteria* sp. have been isolated from camel udders (Zeryehun and Abera, 2017), the highly infective *Streptococcus agalactiae* (group B *Streptococcus*) is the main mastitis-causing bacterium. Our recent studies (Ochieng et al., 2021; Murungi et al., 2022; Maichomo et al., 2023) have identified several bacterial strains implicated in causing camel mastitis in Kenya, predominantly belonging to the*S. agalactiae and Staphylococcus aureus* species. The ubiquity of *S. aureus* as an environmental contaminant and its reported likelihood to cause zoonotic infections (Roy et al., 2024), and the zoonotic ability of *S. agalactiae* (Botelho et al., 2018) will in addition to the probability of amplifying the burden of camel mastitis endanger human health in the ASALs of Kenya that largely lack access to clean water, sanitation and hygiene services.

Due to escalating antimicrobial resistance (AMR) (Naranjo-Lucena and Slowey, 2023), often linked to over- and mis-use of antimicrobials, treatment of camel mastitis is increasingly daunting. This scenario is further complicated by biofilms formation and the ability of mastitis-causing bacteria to penetrate and attach to the mammary epithelium cells (Saeed et al., 2024). Thus, camel mastitis is bound to progressively become non-responsive to conventional antimicrobials. Notably, due to pasture scarcity in the ASALs of Kenya occasioned by prolonged droughts due to adverse impacts of climate change, pastoralists’ camels are likely to transmit antimicrobial resistance genes (ARGs) as they traverse lands in search of pasture. Thus, there is an urgent, pressing need for the development of an effective subunit camel mastitis vaccine to add to the commonly available commercial vaccines and herd-specific auto vaccines based on killed whole bacterial cells used in dairy cows (Ismail, 2017).

Following sequencing and assembly of the genomes of *Staphylococcus aureus*, *Streptococcus agalactiae*, *Lactobacillus lactis*, *Enterococcus faecium* and *Enterococcus gallinarum* strains isolated from mastitis-infected pastoralists’ camels in Kenya, we undertook *in silico* analyses and elucidated several putative antigenic vaccine candidates from which an array of epitopes was uncovered and used to design a potential multi-epitope camel mastitis vaccine.

## 2 Materials and methods

### 2.1 Data retrieval

The proteomes of *S. aureus* (Accession: JARJBG010000027.1), *S. agalactiae* (Accession: JANCLS000000000), *L. lactis* (Accession: JAOPKV000000000), *E. faecium* (Accession: JAOTOH000000000) and *E. gallinarum (*Accession: JAOTOI010000000) were retrieved from the GenBank. Sequencing and assembly protocols are outlined in our recent publications (Ochieng et al., 2021; Murungi et al., 2022; Maichomo et al., 2023). Non-redundant sequences in each proteome were obtained by clustering using CD-HIT (Fu et al., 2012) at a sequence identity cut-off of 90%.

### 2.2 Selection of potential vaccine candidate protein sequences

Antigenicity was set as the core criteria in choosing potential vaccine candidates. The five bacterial proteomes were screened using VaxiJen v2.0 (https://www.ddg-pharmfac.net/vaxijen3/home/) (Doytchinova and Flower, 2007) at a cut-off value of 0.7. Thereafter, the subcellular localization of the selected sequences and presence of signal peptides to differentiate between secretory and non-secretory proteins was determined using PSORTb (https://psort.org/psortb/) (Yu et al., 2010) and SignalP 5.0 server (https://services.healthtech.dtu.dk/services/SignalP-5.0/) (Almagro Armenteros et al., 2019).

### 2.3 Physicochemical characterization of putative vaccine candidates

Transmembrane topology of the transmembrane proteins in the identified set of probable vaccine candidates was ascertained using DeepTMHMM (https://dtu.biolib.com/DeepTMHMM/) (Hallgren et al., 2022). Adhesion-like characteristics and allergenicity of the putative candidates were predicted using SPAAN (Sachdeva et al., 2005) at a cut-off of 0.5 and AllerTOP server (https://www.ddg-pharmfac.net/allertop_test/) (Dimitrov et al., 2014) respectively.

### 2.4 Linear B-cell epitope prediction

The selected antigenic sequences were screened for linear B-cell epitopes using Bepipred Linear Epitope Prediction 2.0 tool (Jespersen et al., 2017) in the Immune Epitope Database and Analysis Resource (IEDB, https://www.iedb.org/). The potential of exposed, non-allergenic epitopes to trigger antibody production was ascertained using IgPred (http://crdd.osdd.net/raghava/igpred/bkp-biology-direct/index.html) (Gupta et al., 2013).

### 2.5 Prediction of cytotoxic T-lymphocyte and helper T-lymphocyte epitopes

Probable CTL epitopes in the candidate protein sequences were uncovered using the NetCTL 1.2 server (https://services.healthtech.dtu.dk/services/NetCTL-1.2/) (Larsen et al., 2007) using default settings (threshold = 0.75). Using artificial neural networks, NetCTL 1.2 integrates prediction of peptide major histocompatibility complex (MHC) class I binding, proteasomal C terminal cleavage and TAP transport efficiency allowing prediction of CTL epitopes restricted to 12 MHC class I supertype. Thereafter, helper T cells 15-mer epitopes were predicted using NetMHCII 2.3 server (https://services.healthtech.dtu.dk/services/NetMHCII-2.3/) (Jensen et al., 2018). Three human HLA-DR class II alleles were evaluated. According to standards, the lowest consensus scores of the peptides presumed to be the best binders have a lower percentile rank indicating higher affinity. An IC50 cut-off of ≤ 50 and percentile rank <1 were set as the selection criteria. The epitopes unveiled were further evaluated for their propensity to induce Th1 type immune response characterized by the production of IFN-gamma using the IFNepitope server (http://crdd.osdd.net/raghava/ifnepitope/) (Dhanda et al., 2013). In this case, 15-mer HTL epitopes were selected based on an IC50 value of ≤50 nM, lowest percentile rank score and highest prediction score which point to avidity of epitope binding (Nielsen et al., 2008).

### 2.6 Construction of a putative multi-epitope camel mastitis vaccine

A multi-epitope potential vaccine was constructed using an array of the predicted CTL and HTL epitopes. The CTL and HTL epitopes were linked using AAY and GPGPG linkers respectively (Rana and Akhter, 2016; Kalita et al., 2019). The long alpha chain of human IL-12 was included as an adjuvant to amplify the immunogenicity of the vaccine construct (Chatterjee et al., 2021). The adjuvant was linked to the initial CTL epitope through an EAAAK linker at the N-terminal of the sequence. A histidine tag was included in the C-terminal of the vaccine construct.

### 2.7 Evaluation of antigenicity, allergenicity, immunogenicity and toxicity

The antigenicity of the candidate vaccine construct was evaluated using VaxiJen v2.0 server while the allergenicity was assessed using AlgPred 2.0 (Sharma et al., 2021) and AllerCatPro (https://allercatpro.bii.a-star.edu.sg/) (Maurer-Stroh et al., 2019).

### 2.8 Physiochemical properties and solubility prediction

The physiochemical properties of the putative vaccine including theoretical isoelectric point (pI), estimated half-life, instability index, molecular weight (M_w_), aliphatic index and grand average of hydropathy (GRAVY) were determined using the ExPASy ProtParam tool (Gasteiger et al., 2003).

### 2.9 Decoding the secondary and tertiary structure of the putative vaccine candidate

The three-dimensional (3-D) structure of the designed camel mastics vaccine candidate was inferred using the AlphaFold3 sever (https://alphafoldserver.com/) (Abramson et al., 2024) and the robustness of the model ascertained by determining the Ramachandran plot on the ExPASy server (https://swissmodel.expasy.org/).

### 2.10 Molecular docking of the vaccine construct onto TLR-2

To determine the potential of the vaccine candidate to trigger innate immune responses, blind docking of the putative vaccine candidate onto TLR-2 (PDB ID:5D3I) was performed using the ClusPro server (https://cluspro.org/login.php) (Kozakov et al., 2017). Analysis of the non-covalent interactions stabilizing the interface between the vaccine candidate and TLR-2 was performed using COCOMAPS 2.0 (https://aocdweb.com/BioTools/cocomaps2/) (Chawla et al., 2025). Toll-like receptors activate immune responses by recognizing pathogen-associated molecular pattern molecules (PAMPs) on bacteria, viruses, fungi and parasites. The docked complex was visualized using PyMOL (Schrodinger and DeLano, 2020).

### 2.11 Normal mode analysis

To further delineate the interaction between the potential vaccine candidate and TLR-2, normal mode analysis was undertaken using the iMODS server (https://imods.iqf.csic.es/) using a timescale of 10 nanoseconds (Lopéz-Blanco et al., 2011).

### 2.12 Simulation of the host immune response

Prediction of how the vaccine candidate would elicit an immune response was conducted using the C-ImmSim server (https://kraken.iac.rm.cnr.it/C-IMMSIM/index.php) which is underpinned by the Celada-Seiden algorithm that models immune reactions in a vaccinated mammalian system (Rapin et al., 2010). In the simulations, apart from the assumption that the candidate vaccine would be administered without a lipopolysaccharide (LPS) adjuvant, all other parameters were at a default setting. The sigma-70 factor of RNA polymerase subunit was used as the negative control antigen with the measurement of immune heterogeneity undertaken and interpreted using the Simpson index D (Dolley et al., 2023).

**Figure 1:**
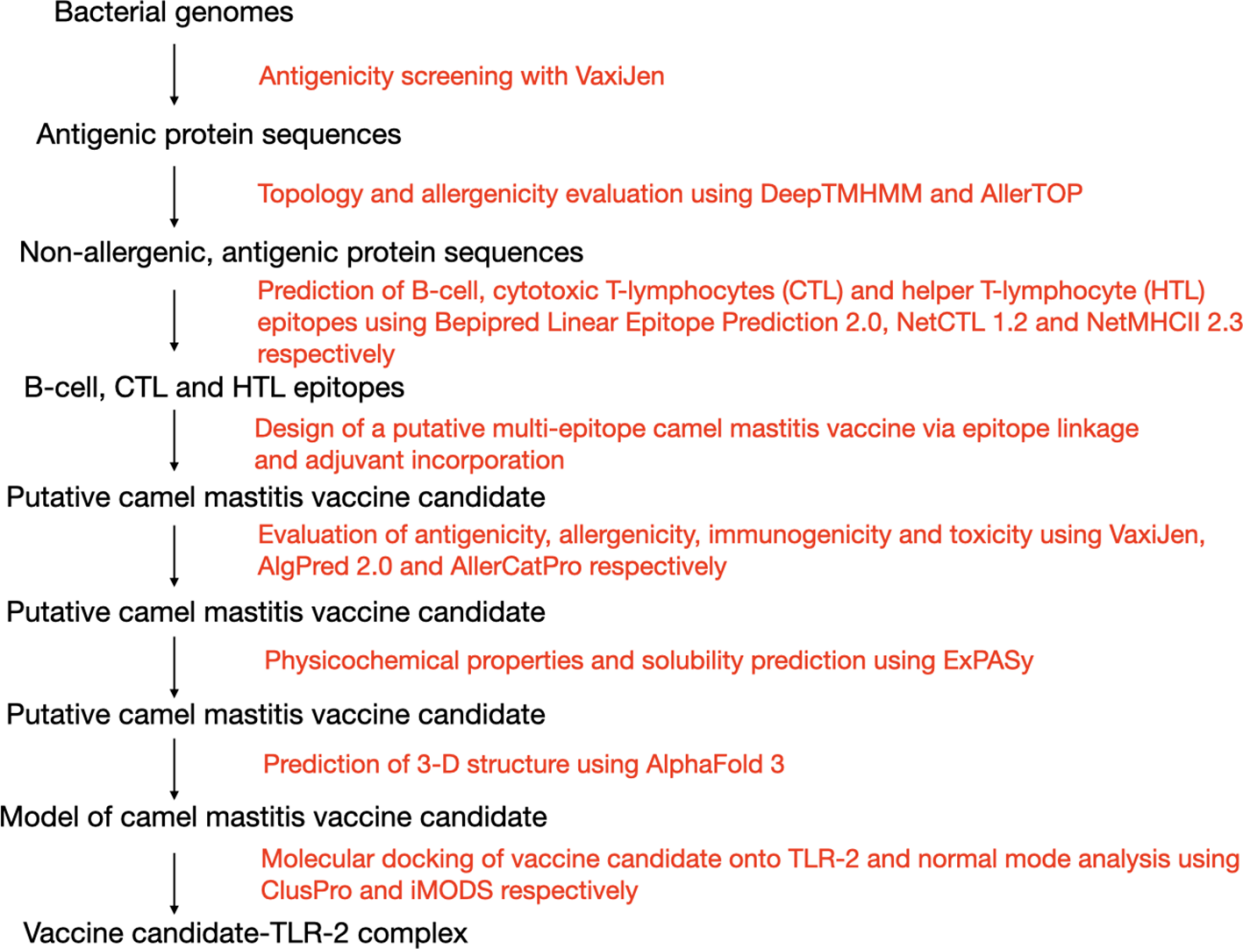
The proteomic analysis pipeline used in the design of a camel mastitis multi-epitope vaccine candidate. The parameter being evaluated and the software used are indicated in red.

In sum, the proteomic analysis pipeline is illustrated in **Figure 1**.

## 3 Results

### 3.1 Identification of potential vaccine candidates

Analyses of the subtracted proteomes of *S. aureus, S. agalactiae, L. lactis, E. faecium* and *E. gallinarum* using VaxiJen v2.0 revealed an array of highly scoring (>0.7) putative antigenic sequences. Specifically, the initial number identified antigenic proteins in *S. aureus*, *S. agalactiae*, *L. lactis*, *E. faecium* and *E. gallinarum* were 125, 29, 103, 58 and 77 respectively. These sequences were subsequently parsed based on the cellular localization with only the top nineteen membrane localized and secreted proteins chosen for further downstream analyses (**Table 1**). The physicochemical properties of the antigenic proteins are outlined in **Supplementary Table 1**. Notably, in selecting sequences for downstream analyses, we focused on *S. aureus* and *S. agalactiae* that have been reported as the principal etiological agents of mastitis in the field (Seligsohn et al., 2020). The sequences selected as potential vaccine candidates included PDZ domain-containing protein, YpmS family protein, cell wall synthase accessory phosphoprotein MacP, pathogenicity island protein and VraH family protein.

**Table 1:** A depiction of the top VaxiJen predicted antigenic S. aureus and S. agalactiae proteins.

Bacterial source	GenBank accession	Protein name	VaxiJen score	Subcellular localization	Transmembrane helices	AllerTOP prediction	Signal peptide
*S. aureus*	MDF3296226.1	Hypothetical protein P3G69_09200	1.4155	Cytoplasmic membrane	1	Probable non-allergen	No
*S. aureus*	MDF3345085.1	Cell surface protein, partial	1.4003	Extracellular	1	Probable non-allergen	No
*S. aureus*	MDF3296234.1	Hypothetical protein P3G69_09240	1.3336	Cytoplasmic membrane	1	Probable non-allergen	No
*S. aureus*	MDF3296909.1	DUF4887 domain-containing protein	1.1962	Cytoplasmic membrane	1	Probable non-allergen	No
*S. aureus*	MDF3294667.1	Elastin-binding protein EbpS	1.136	Cytoplasmic membrane	1	Probable non-allergen	No
*S. aureus*	MDF3294497.1	Hypothetical protein P3G69_00220	1.028	Extracellular	0	Probable non-allergen	No
*S. aureus*	MDF3295037.1	Type 1 toxin-antitoxin system Fst family toxin	0.9862	Cytoplasmic membrane	1	Probable non-allergen	No
*S. aureus*	MDF3296810.1	Pathogenicity island protein	0.9513	Cytoplasmic membrane	1	Probable non-allergen	No
*S. agalactiae*	MCP9190290.1	Cell wall synthase accessory phosphoprotein MacP	0.9461	Cytoplasmic membrane	1	Probable non-allergen	No
*S. aureus*	MDF3295131.1	Hypothetical protein P3G69_03545	0.9448	Extracellular	1	Probable non-allergen	No
*S. aureus*	MDF3343883.1	Hypothetical protein P3G69_08555	0.9351	Extracellular	1	Probable non-allergen	No
*S. aureus*	MDF3294606.1	Hypothetical protein P3G69_00775	0.9042	Cytoplasmic membrane	0	Probable non-allergen	No
*S. aureus*	MDF3296979.1	VraH family	0.8981	Cytoplasmic membrane	1	Probable non-allergen	No
*S. aureus*	MDF3294509.1	Preprotein translocase subunit YajC	0.8864	Cytoplasmic membrane	1	Probable non-allergen	No
*S. aureus*	MDF3294841.1	Hypothetical protein P3G69_02025	0.8767	Extracellular	0	Probable non-allergen	No
*S. aureus*	MDF3295053.1	Putative metal homeostasis protein	0.867	Extracellular	0	Probable non-allergen	No
*S. aureus*	MDF3296041.1	Hypothetical protein P3G69_08260	0.8409	Cytoplasmic membrane	1	Probable non-allergen	No
*S. agalactiae*	MCP190898.1	YpmS family protein	0.8236	Cytoplasmic membrane	1	Probable non-allergen	No
*S. agalactiae*	MCP9190874.1	PDZ domain-containing protein	0.8131	Cytoplasmic membrane	0	Probable non-allergen	No

Allergenicity, subcellular localisation, signal peptide presence or absence and the number of transmembrane helices for the candidates are also indicated.

### 3.2 B and T cell epitope mapping

#### 3.2.1 B cell epitopes

The Bepipred Linear Epitope Prediction 2.0 tool within the IEDB server was used to determine B cell epitopes in the prioritized antigenic sequences (**Table 2**). Bepipred 2.0 predicts B-cell epitopes in a protein sequence using a Random Forest algorithm trained on detecting epitopes and non-epitopes in crystal structures of antibody-antigen complexes and outputs a prediction score for each amino acid in the input sequence whereby residues with scores above the threshold (default value is 0.5) are predicted to be part of an epitope.

**Table 2:** Predicted linear B cell epitope sequences identified in the prioritised *S. aureus* and *S. agalactiae* antigenic protein sequences.

Bacterial source	GenBank	Protein name	Sequence length (aa)	Predicted antigenic peptide	Peptide length (aa)	Average bepipred score
*S. aureus*	MDF3296226.1	hypothetical protein P3G69_09200	30	NAYSH	5	0.4
*S. aureus*	MDF3345085.1	cell surface protein, partial	284	GQPAVTTGEQAGGKPAATKPEGASAETAPAPNNADENHAQPSPQTGGTTATQAGLVKPSGDSTPTGEPNKADDQGTQIKPTSNQGTTATTNETGNQKPSGQTGNTENTPNDGTQLKPNNDPAVTGTPNTNGEQTGQPNATVTPGQGNEEINGASKPGEVSPKPEENNPTATEPGTTAPGNTNQDTQVKPNTDQTATGTPAGTDNQNTQQGNTEQNNQNAQPSAPGTTDQAGATVKPSTTPNQDAEVKPNPDQNNTPTNGQDGNQTGQGTQVKPNNDQ	277	0.594
*S. aureus*	MDF3296234.1	hypothetical protein P3G69_09240	66	ERKRTKKKQLEKEKANTLNQNTNDTESSNQEPSLQQAKEQK	41	0.501
*S. aureus*	MDF3296909.1	DUF4887 domain-containing protein	209	HSNKAKERMLNEQKQEQKEKRQKENAEKERKKKQQEEKEQNELDSQANQYQQLPQQNQYQYVPPQQQAPTKQRPAKEENDDKASKDESKDKDDKASQDKSDDNQKKTDDNKQPAQPKPQPQQPTPKPNNNQQNNQSNQQAKPQAPQQNSQSTTNKQNN	158	0.569
*S. aureus*	MDF3294667.1	elastin-binding protein EbpS	486	NFKDDFEKNRQSIDTNSHQDHTEDVEKDQSELEHQDTIENTEQQFPPRNAQRRKRRRDLATNHNKQVHNESQTFEDNVQNEAGTIDDRQVESSHSTESQEPSHQDSTPQHEEGYYNKNAFAMDKSHPEPIEDNDKHETIKEAENNTEHSTVSDKSEAEQSQQPKPYFATGANQANTSKDKHDDVTVKQDKDESKDHHSGKKGAAIGAGTAGVAGAGAMGVSKAKKHSNDAQNKSNSGKVNNSTEDKASEDKSKEHHNGKKGAAICAGTAGLAGGAASNSASAASKPHASNNASQNNDEHDHHDRDKERKKGGMAKVLLPLIAAVLIIGALAIFGGMALNNHNNGTKENKIANTNKNNADESKDKDTSKDASKDKSKSTDSDKSKDDQDKATKDESDNDQNNANQANNQAQNNQNQQQANQNQQQQQQRQGGGQRHTVNGQEN	443	0.595
*S. aureus*	MDF3294667.1	elastin-binding protein EbpS	486	YGSGSPENVEKIRRANGLSGNNIRNGQQ	28	0.595
*S. aureus*	MDF3294497.1	hypothetical protein P3G69_00220	39	SKQKQANEQQKAQNLFA	17	0.539
*S. aureus*	MDF3294497.1	hypothetical protein P3G69_00220	39	RQLQNSNSESSND	13	0.539
*S. aureus*	MDF3295037.1	type 1 toxin-antitoxin system Fst family toxin	35	TRNNKK	6	0.402
*S. aureus*	MDF3296810.1	pathogenicity island protein	48	TGG	3	0.358
*S. agalactiae*	MCP9190290.1	Cell wall synthase accessory phosphoprotein MacP	80	SNRGEKVSGQTILDQETKIISTEDGMEQLTDENGKHIYKSRRIENAKRNEFQR	53	0.494
*S. aureus*	MDF3295131.1	hypothetical protein P3G69_03545	61	NKIDESDTHL	10	0.474
*S. aureus*	MDF3295131.1	hypothetical protein P3G69_03545	61	IMSKKVYNMNVKTNF	15	0.474
*S. aureus*	MDF3343883.1	hypothetical protein P3G70_08555	61	NKIDESDTHL	10	0.480
*S. aureus*	MDF3343883.1	hypothetical protein P3G70_08555	61	IMSKKVYNMNVKTNF	15	0.480
*S. aureus*	MDF3343883.1	hypothetical protein P3G70_08555	61	CEVRR	5	0.480
*S. aureus*	MDF3294606.1	hypothetical protein P3G69_00775	48	SQYSLKLK	8	0.464
*S. aureus*	MDF3294606.1	hypothetical protein P3G69_00775	48	LYNIETDY	8	0.464
*S. aureus*	MDF3294606.1	hypothetical protein P3G69_00775	48	EGKT	4	0.464
*S. aureus*	MDF3296979.1	VraH family protein	73	NDLLNKKWSLE	11	0.455
*S. aureus*	MDF3296979.1	VraH family protein	73	KDDDDVDEIAEKYDYQDE	18	0.455

The length, amino acid composition and overall Bepipred Linear Epitope Prediction 2.0 score are indicated.

#### 3.2.2 T cell (HTL and CTL) epitopes

MHC-1 molecules are expressed on the cell surface of all nucleated cells and serve to present peptide fragments derived from intracellular proteins to the immune system, alerting it to virally infected cells and subsequently triggering virus specific cytotoxic T lymphocytes (CTL) response (Hewitt, 2003). Collectively, 56 CTL epitopes were identified from several of the antigenic proteins under investigation using the NetCTL 1.2 server which predicts CTL epitopes in protein sequences (**Table 3**). Each predicted CTL epitope was assigned a particular score based on the prediction algorithm with only the top ten scoring motifs (indicating higher MHC I-binding affinity) considered for incorporation in the candidate vaccine following determination of their allergenicity and toxicity. Likewise, the NetMHCII 2.3 server was used for the prediction of 15-mer HTL epitopes from the selected antigenic proteins against a set of three human HLA alleles (HLA- DRB1*01:01, HLA- DRB1*01:03 and HLA- DRB1*03:01). NetMHCII 2.3 output gives the affinity (nM) and strength of epitope binding (strong binding (SB) and weak binding (WB) to MHC-II molecules. HTL epitopes with strong binding (SB) to MHC-II molecules are shown in **Table 4**.

**Table 3:** CTL epitopes predicted from the *S. aureus* and *S. agalactiae* antigenic protein sequences using the NetCTL 1.2 server.

GenBank ID	CTL epitope sequence	Predicted MHC binding affinity (nM)	Prediction score
MDF3296226.1	YSHASLLFF	0.2692	1.2895
MDF3296226.1	HASLLFFIY	0.4313	2.0853
MDF3296226.1	FIYLLFIMY	0.1740	0.1740
MDF3345085.1	TTATQAGLV	0.3573	1.6728
MDF3345085.1	NTDQTATGT	0.2062	0.8309
MDF3345085.1	GTDNQNTQQ	0.2411	1.0511
MDF3345085.1	TTDQAGATV	0.4317	1.9765
MDF3296909.1	ELDSQANQY	0.5579	2.6500
MDF3294667.1	MSNNFKDDF	0.1630	0.8870
MDF3294667.1	STPQHEEGY	0.2695	1.3386
MDF3294667.1	TVNGQENLY	0.2737	1.4430
MDF3294667.1	NLYRIAIQY	0.1094	0.7613
MDF3295037.1	LIDIMTSAL	0.1464	0.8052
MDF3295037.1	MTSALSGCL	0.1616	0.7722
MDF3295037.1	TSALSGCLV	0.1760	0.7824
MDF3296810.1	LLLAISNMY	0.2302	1.2480
MDF3296810.1	NMYVAFSVY	0.1381	0.8960
MCP9190290.1	STEDGMEQL	0.1593	0.8545
MCP9190290.1	LTDENGKHI	0.1837	0.9075
MCP9190290.1	LILLALLFY	0.1832	1.0400
MDF3295131.1	DESDTHLSY	0.1156	0.7602
MDF3295131.1	ESDTHLSYK	0.1822	0.7988
MDF3295131.1	MMMKACYSY	0.1983	1.1116
MDF3295131.1	YSYFFIFSY	0.3892	1.9550
MDF3343883.1	DESDTHLSY	0.1156	0.7609
MDF3343883.1	ESDTHLSYK	0.1822	0.7978
MDF3343883.1	MVMKACYSY	0.1487	0.8949
MDF3343883.1	YSYFFIFSY	0.3892	1.9550
MDF3294606.1	LTAIYFSIF	0.3782	1.7834
MDF3294606.1	YISQYSLKL	0.1489	0.8254
MDF3294606.1	YSLKLKTLY	0.4952	2.3824
MDF3294606.1	TLYNIETDY	0.1187	0.8076
MDF3294606.1	ETDYNYKIV	0.2164	0.9621
MDF3296979.1	SLEDLFWLI	0.1675	0.8597
MDF3296979.1	VIAYFFFFY	0.4399	2.1647
MDF3296979.1	DVDEIAEKY	0.4872	2.3352
MDF3294509.1	IVVIFAVMY	0.1572	0.9639
MDF3294841.1	RSENMNMKK	0.1448	0.7759
MDF3296041.1	QAIANVTFY	0.2086	1.1659
MCP910898.1	FTAVIASRL	0.1864	0.9362
MCP910898.1	QLNKTIALY	0.3386	1.7274
MCP910898.1	TIALYIKQL	0.1221	0.8128

**Table 4:** HTL epitopes from the antigenic sequences predicted by NetMHCII-2.3 to bind to MHC class II molecules.

GenBank ID	Alleles	HTL epitope sequence	Affinity (nM IC50)	Binding level
MDF3294606	DRB1_0101	SIFTFYISQYSLKLK	4.9	SB
MDF3294606	DRB1_0101	IFTFYISQYSLKLKT	4.0	SB
MDF3294606	DRB1_0101	FTFYISQYSLKLKTL	3.6	SB
MDF3294606	DRB1_0101	TFYISQYSLKLKTLY	4.2	SB
MDF3294606	DRB1_0101	FYISQYSLKLKTLYN	6.4	SB
MDF3294509	DRB1_0101	AVMYFLMIRPQQKRA	8.0	SB
MDF3294509	DRB1_0101	VMYFLMIRPQQKRAK	6.9	SB
MDF3294509	DRB1_0101	MYFLMIRPQQKRAKQ	8.1	SB
MDF3296041	DRB1_0101	DATVYVSLPLVQAIA	70	SB
MDF3296041	DRB1_0101	ATVYVSLPLVQAIAN	6.4	SB
MDF3296041	DRB1_0101	TVYVSLPLVQAIANV	7.3	SB
MCP9190898	DRB1_0101	LAINLSFTAVIASRL	7.2	SB
MCP9190898	DRB1_0101	AINLSFTAVIASRLI	4.5	SB
MCP9190898	DRB1_0101	INLSFTAVIASRLIQ	3.8	SB
MCP9190898	DRB1_0101	NLSFTAVIASRLIQV	3.5	SB
MCP9190898	DRB1_0101	LSFTAVIASRLIQVR	3.8	SB
MCP9190898	DRB1_0101	SFTAVIASRLIQVRE	4.6	SB
MCP9190874	DRB1_0101	TYKGVYVLNLAKNST	7.3	SB
MCP9190874	DRB1_0101	YKGVYVLNLAKNSTF	5.4	SB
MCP9190874	DRB1_0101	KGVYVLNLAKNSTFK	4.2	SB
MCP9190874	DRB1_0101	GVYVLNLAKNSTFKD	5.4	SB
MCP9190874	DRB1_0101	VYVLNLAKNSTFKDR	6.6	SB
MCP9190874	DRB1_0101	SQLIKYVAALHLGDK	7.6	SB
MCP9190874	DRB1_0101	QLIKYVAALHLGDKV	8.1	SB
MDF3294841	DRB1_0103	MESIFKIKIELMNVI	2329.6	SB
MDF3294841	DRB1_0103	ESIFKIKIELMNVIC	1980	SB
MDF3294841	DRB1_0103	SIFKIKIELMNVICR	1724.9	SB
MDF3294841	DRB1_0103	IFKIKIELMNVICRS	2126.5	SB
MDF3294841	DRB1_0103	FKIKIELMNVICRSE	2551	SB
MDF3294841	DRB1_0103	KIKIELMNVICRSEN	2647.3	SB
MDF3295053	DRB1_0103	PNIKTRKRALKIIKQ	2677	SB
MDF3295053	DRB1_0103	IKTRKRALKIIKQHK	2559	SB
MCP9190874	DRB1_0103	FLLLVLASLVVRLPY	2698.5	SB
MDF3295053	DRB1_0301	MKLDLQTARRNLNSP	38.4	SB

The strongly binding HTL epitopes (**Table 4**) were evaluated for their ability to stimulate the production of interferon-γ using the IFNepitope server which predicts IFN-γ versus non-IFNγ epitopes using SVM and motif-based hybrid analysis. Seven peptides (**Table 5**) were revealed as potentially capable of triggering interferon-γ production. Thereafter, the seven peptides were assessed for their ability to stimulate IL-4 and IL-10 synthesis using the IL4pred and IL10pred servers, respectively. Both servers are underpinned by a SVM classifier that considers amino acid composition, dipeptide composition, amino acid propensity, and physicochemical properties as input parameters (Dhanda et al., 2013). Indeed, the seven interferon-γ producing epitopes were predicted to trigger the production of IL-4 and IL-10 and thus appropriate for incorporation in the vaccine construct.

**Table 5:** HTL epitopes identified to potentially induce interferon-γ production.

GenBank accession	HTL epitope sequence	Method	Result	Score
MDF3294606	TFYISQYSLKLKTLY	SVM	Positive	0.10497891
MDF3294606	FYISQYSLKLKTLYN	SVM	Positive	0.20272651
MDF3294509	AVMYFLMIRPQQKRA	SVM	Positive	0.16142613
MDF3294509	VMYFLMIRPQQKRAK	SVM	Positive	0.11507428
MDF3296041	DATVYVSLPLQAIA	SVM	Positive	0.53491282
MDF3296041	ATVYVSLPLVQAIAN	SVM	Positive	0.2108538
MDF3296041	TVYVSLPLVQAIANV	SVM	Positive	0.3412636

### 3.3 Construction of a candidate multi-epitope camel mastitis vaccine

**Figure 2:**
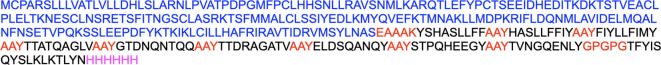
Amino acid sequence of the multi-epitope camel mastitis vaccine candidate. Vaccine adjuvant sequence is depicted in blue, CTL and HTL epitopes are rendered in black, linkers (EAAAK, AAY and GPGPG) are shown in red while the His-tag is illustrated in magenta.

The multi-epitope vaccine was designed by combining the sequences of 9 CTL epitopes predicted to bind to MHC-I with high affinity and 1 interferon-γ inducing HTL epitope selected based in its MHC-II binding affinity and cytokine-inducing ability. The CTL and HTL epitopes were joined using AAY and GPGPG linkers, respectively. To ensure that the vaccine candidates elicits a strong immune response, an adjuvant (219 amino acids long alpha chain of human IL-12; Uniprot ID: P29459) was added to the N-terminal of the construct using the EAAK linker (Afonso et al., 1994; Bhattacharjee et al., 2023). Moreover, to enable affinity purification following protein expression, a 6XHis tag was fused to the C-terminal of the vaccine construct. After the incorporation of the adjuvant, linkers and histidine tag, the final length of the candidate vaccine was 353 amino acids (**Figure 2**).

### 3.4 Evaluation of antigenicity, allergenicity, immunogenicity and toxicity

Determination of the antigenicity of the vaccine construct using VaxiJen returned a score of 0.5596 against a threshold of 0.4 indicating that the candidate is potentially capable of eliciting host immune response. Evaluation of allergenicity using AlgPred 2.0 and AllerCatPro did not reveal any evidence of sequence or structural similarity with known allergens indicating that the vaccine candidate is plausibly non-allergenic.

### 3.5 Physiochemical properties and solubility of the putative vaccine candidate

Using ProtParam, the average molecular weight of the vaccine construct was predicted to be 39718.43 with a theoretical isoelectric point (pI) of 6.15. Moreover, the candidate’s instability index (II) was 37.30 which classifies the protein as stable (II<40 indicates protein stability). The estimated *in vitro* half-life in mammalian reticulocytes and Escherichia coli was >30 h and >10 h, respectively indicating *in vivo* stability of the putative vaccine candidate. The candidate had an aliphatic index and grand average of hydropathicity (GRAVY) of 87.48 and -0.071 respectively, indicating that thermostability and aqueous solubility.

### 3.6 Secondary and tertiary structure features of the vaccine candidate

**Figure 3:**
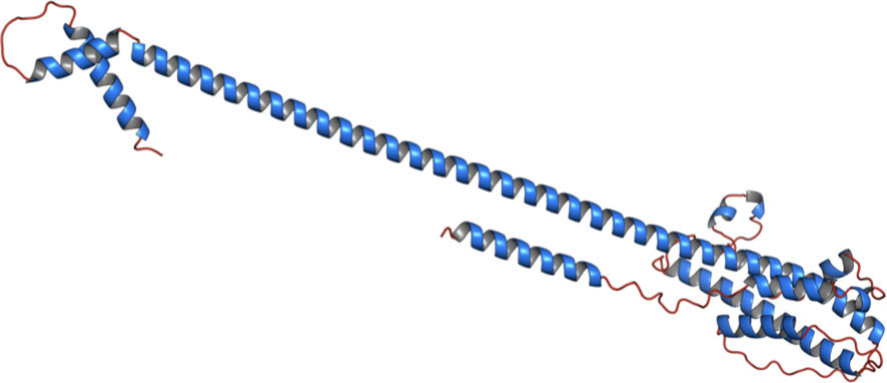
Cartoon representation of the predicted three-dimensional (3-D) model of the camel mastitis vaccine candidate. The model predicted using the AlphaFold server had a predicted template modelling (pTM) score of 0.52. A pTM score above 0.5 means the overall predicted fold for the protein is likely similar to the true structure. Helices and loops are coloured marine and red respectively and highlighted in grey50.

The 3-D model of the putative vaccine candidate inferred using AlphaFold 3 revealed it to contain 11 helices linked by loops (**Figure 3**). The model had a predicted template modelling (pTM) score of 0.52. A pTM score above 0.5 means the overall predicted fold for the protein is likely similar to the true structure. Moreover, evaluation of the structural robustness of the model using the Ramachandran plot revealed that 93.94% of the residues were in the favored regions, 6.06% in the allowed regions and 0% in the outlier regions.

### 3.7 Molecular docking of the vaccine construct onto TLR-2

**Figure 4:**
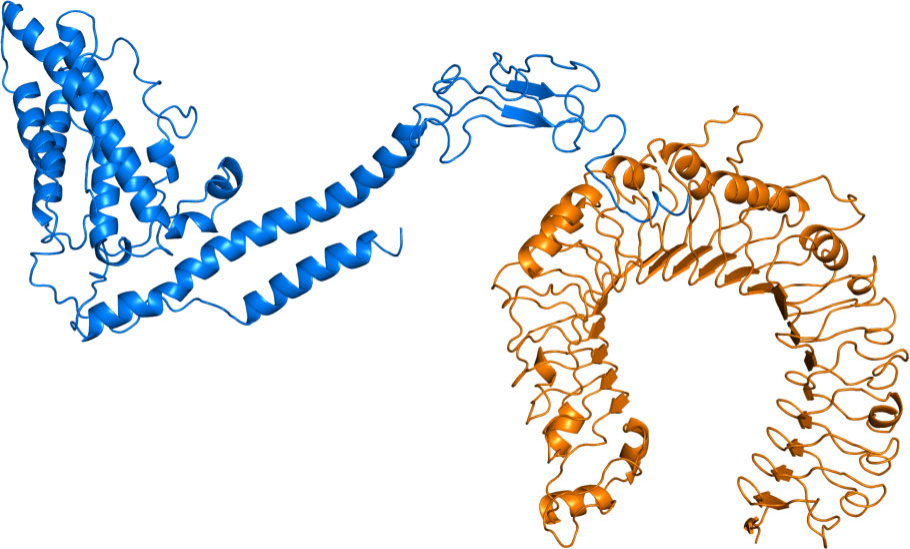
Depiction of the interaction between the designed vaccine candidate (coloured marine and highlighted in grey50) with TLR-2 (coloured orange and highlighted in grey50) as predicted using the protein-protein docking program ClusPro. The candidate interacts with TLR-2 via a C-terminal motif.

The CASTp server identified various binding sites in the vaccine construct with the largest having a binding area and volume of 326.094 Å2 and 244.195 Å3 respectively. However, ClusPro server predicted the interaction with TLR-2 (-31.36 kJ/mol) to plausibly occur via a much smaller pocket in the C-terminal region of the construct (**Figure 4**). Analysis of the non-covalent interactions stabilizing the interface between the vaccine candidate and TLR-2 revealed weak H-bonds (CH-ON bonds) contacts between ALA121 and LEU324; SER125 and TY323. Moreover, a water-mediated contact was predicted between SER125 and TY323. Activation of the membrane-bound receptor TLR-2, the most indiscriminate of the TLRs with respect to bacteria, virus, parasites and fungi derived pathogen-associated molecular patterns (PAMPs), triggers the production of nuclear factor-kappa B and cytokine that subsequently stimulate innate immunity (Akira et al., 2006).

### 3.8 Normal mode analysis of the vaccine candidate-TLR-2 complex

**Figure 5:**
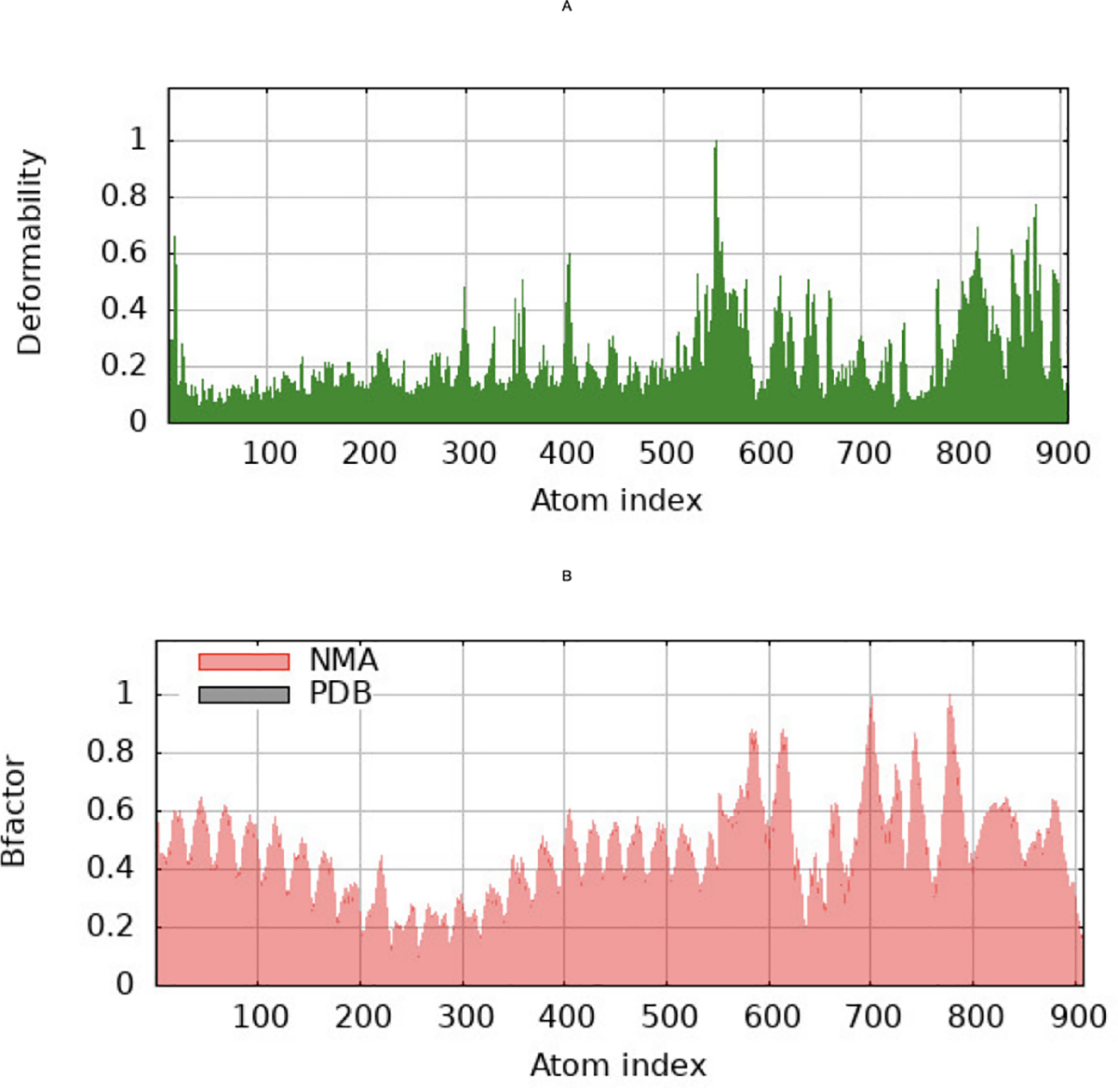
An illustration of deformability **(A)** and B-factor **(B)** of the designed vaccine candidate -TLR-2 complex ascertained using the iMODS server. The main-chain deformability is a measure of the ability of a given molecule to deform at each of its residues. The myriad of deformable regions seen point to the flexibility of the complex.

The iMODS server was used to explore the collective motions of the vaccine construct-TLR-2 complex and revealed an overall Eigenvalue of 5.155235e-07 for the complex. Notably, the deformability (**Figure 5A**) and B-factor ((**Figure 5B**) plots indicate that the complex is adequately flexible.

### 3.9 Simulation of the ability of the vaccine construct to elicit host immune response

**Figure 6:**
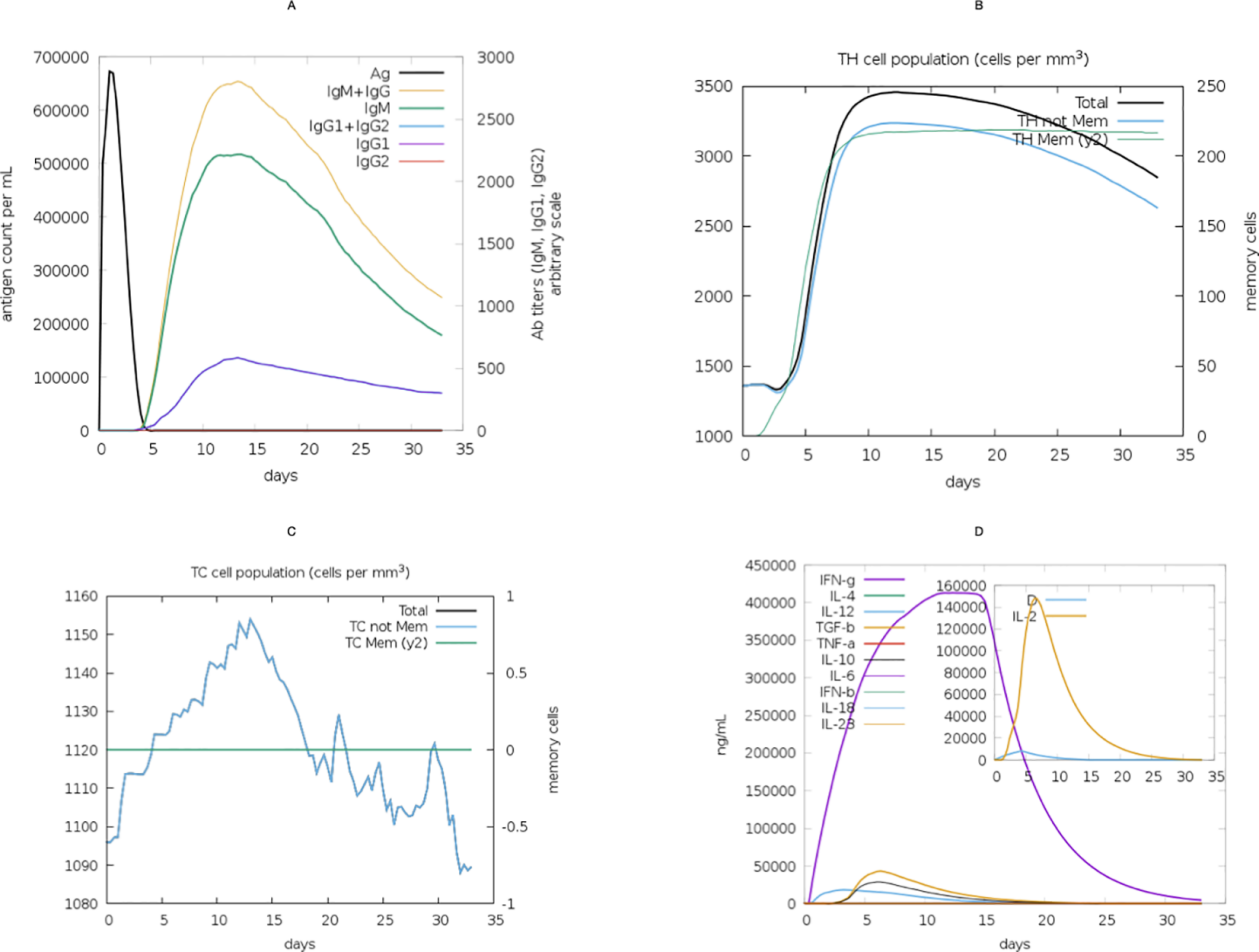
Depiction of the simulated ability of the vaccine candidate to broadly activate the host immune response **(A)**, production of T-helper lymphocytes **(B)**, T-cytotoxic lymphocytes **(C)**, cytokines and interleukins **(D)**. Altogether, the vaccine candidate is thus likely to trigger an immune response.

The vaccine construct was subjected to *in silico* immune simulations to ascertain its potential to stimulate the adaptive immune system. The results obtained indicate that it is capable of inducing production of high levels of IgG and IgM antibodies with a corresponding reduction in antigen levels (**Figure 6A**). Additionally, it was shown to be capable of triggering synthesis of T-helper lymphocytes (**Figure 6B**), cytotoxic T lymphocytes (**Figure 6C**) and, cytokines and interleukins (**Figure 6D**). In sum, the designed vaccine construct would potentially elicit a strong immune response.

## 4 Discussion

Camel mastitis (inflammation of the mammary gland) causes immense socio-economic losses for pastoral and nomadic communities in the arid and semi-arid (ASALs) regions of Africa where the one-humped camels (*Camelus dromedarius*) are an invaluable source of livelihood and nutrition due to their resilience to severe droughts that cause high cattle, sheep and goat mortality (Mwangi et al., 2022). Specifically in the ASALs of Kenya, camel productivity is severely threatened by severe droughts linked to climate change and mastitis which endanger the livelihoods and nutrition of pastoralists in these regions.

The importance of camels in buttressing economic and food security for pastoralists in these regions is bound to become increasingly entrenched by the confluence of the adverse impacts of climate change and extensive dissemination of antimicrobial resistance (AMR) that cause frequent severe droughts and render current antimicrobials largely ineffective respectively. Notably, unlike in other dairy animals where treatment of mastitis with intra mammary antibiotics is effective, this approach is very challenging in camels due to the unique anatomy of camelidae udder (Aqib et al., 2018). Therefore, there is a pressing need for the development of a novel, efficacious camel mastitis vaccine. Indeed, the Kenya AMR Policy and AMR Action Plan recommend the use of vaccines as a sustainable disease control option. In the One-Health framework, reverse vaccinology is a particularly attractive strategy for accelerating the development of human and livestock vaccines in resource limited regions given its ability to significantly shorten development timelines and thus lower cost (Dzayee et al., 2022). Specifically, as illustrated in **Figure 1**, using this methodology, potential immunogenic, non-allergenic and non-toxic candidates are rapidly predicted and prioritized for experimental evaluation.

In this study, we have designed and computationally evaluated a novel, multi-epitope vaccine candidate for camel mastitis comprised of 9 CTL MHC-1 binding epitopes and 1 interferon-γ inducing HTL epitope (**Figure 2**) obtained from selected antigenic protein sequences of local isolates of *S. agalactiae* and S. *aureus* (Murungi et al., 2022; Maichomo et al., 2023), the predominant camel mastitis causing bacteria (Younan et al., 2001; Seligsohn et al., 2021a, Seligsohn et al., 2021b). *In silico* simulations revealed the vaccine construct as capable of strongly stimulating the host innate and adaptive immune responses as evidenced by the elevated synthesis of IgM, IgG, cytokines, macrophages, dendritic cells and natural killer cells (**Figure 6**). As depicted in **Table 1**, the antigenic proteins from which the epitopes were derived are largely cytoplasmic membrane proteins. Surface immunogenic proteins have been demonstrated to be potent inducers of immune responses against microbial infections (Diaz-Dinamarca et al., 2020). For example, iron-regulated surface protein A (IsdA) and surface immunogenic protein (Sip) have been proposed as vaccine candidates for bovine mastitis vaccine (Chatterjee et al., 2021; Pathak et al., 2022). The antigenic proteins from which the epitopes used in the design of the candidate vaccine include *S. aureus* elastin binding protein (EbpS), type I toxin-antitoxin system Fst family toxin, pathogenicity island protein, cell wall synthase accessory phosphoprotein MacP, VraH family protein, preprotein translocase subunit YajC, YpmS family protein and PDZ domain-containing protein. EbpS is an integral membrane protein that plays a key role in the virulence of *S. aureus* together with other factors including cytolytic toxins, superantigens and extracellular proteins (Downer et al., 2002). Specifically, EbpS is an adhesin that binds to the host extracellular matrix component elastin (Campoccia et al., 2009). Another candidate protein, type I toxin-antitoxin system Fst family toxin, detoxifies toxins produced by bacteria and by so doing ensures that bacterial replication, translation and cell division proceed (Masachis and Darfeuille, 2018). On the other hand, the membrane-anchored cell wall synthase accessory phosphoprotein MacP, a cofactor of PBP2a (a penicillin binding protein (PBP) synthase) has been shown to activate the cell wall synthase PBP2a of *Streptococcus pneumoniae* in a phosphorylation-dependent manner (Fenton et al., 2018) thus contributing to cell growth and division. The small staphylococcal transmembrane protein VraH forms, together with VraDE, a three-component system that plays a role in antibiotic resistance and pathogenicity (Popella et al., 2016).

Given that cell-mediated immune response is the principal mode through which the host counters mastitis, the designed vaccine candidate comprised of 9 CTL MHC-1 binding epitopes and 1 interferon-γ inducing HTL epitope. Linking of the CTL epitopes was via AAY while the HTL epitope was linked using a GPGPG linker (**Figure 2**). Moreover an adjuvant (alpha chain of human IL-12) was incorporated at the N-terminal end of the construct. The construct is predicted to be non-allergenic, non-toxic, highly stable and soluble. PRoSA evaluation revealed the 3-D model structure of the designed vaccine construct to be of robust quality. Moreover, the model was shown to be adequately flexible therefore capable of interaction with the immune system components. *In silico* immune simulations indicated the potential vaccine candidate as not only capable of inducing production of high levels of IgG and IgM with a corresponding reduction in antigen levels but also triggers synthesis of T-helper lymphocytes, T-cytotoxic lymphocytes and, cytokines and interleukins.

While our predictive analyses have uncovered a multi-epitope camel mastitis vaccine candidate with desirable characteristics, a potential pitfall that may hamper its development is minimal or no expression of the heterologous protein in *Escherichia coli*. To enable the cloning process, the designed vaccine sequence ought to be codon optimized for prokaryotic expression systems such as the widely used *E. coli* strain K12. In addition, to ensure effective translation of the designed vaccine gene, rho-dependent transcription termination sites, prokaryotic ribosome binding sites and restriction endonuclease cutting sites ought to be avoided (Jiang et al., 2024). Upon successful expression and purification, the vaccine candidate will have to be evaluated for efficacy in mice models whereby specific antibodies ought to be detected in the sera on the immunized mice. An additional putative drawback of the designed vaccine candidate is the potential antigenic variability among diverse field isolates which would constrain the protection conferred by the vaccine. Moreover, given the frequent movement of pastoralists and their animals, vaccine deployment in such settings may be challenging. However, the devolution of veterinary services to counties in Kenya has eased tracking of pastoralists’ animals for vaccination.

Altogether, our study that has uncovered a multi-epitope camel mastitis vaccine candidate significantly contributes to recent broader efforts aimed at developing efficacious multi-epitope livestock vaccines using an immunoinformatics approach that aids in shortening the development timelines and lowering production costs (Abid et al., 2024; Long et al., 2024; Kolla et al., 2024; Wu et al., 2024; Yin et al., 2023).

## 5 Conclusion

Collectively, we have designed and computationally evaluated a camel mastitis candidate vaccine that exhibits desirable properties (immunogenic, non-allergenic and non-toxic) and warrants further experimental investigations. This candidate was derived from antigenic 20 antigenic *S. aureus* and *S. agalactiae* proteins (**Table 1**) prioritized following antigenic screening. Efficacious livestock vaccines offer a viable strategy of combating AMR which threatens the effective prevention and treatment of an ever-increasing range of animal infections. This is particularly relevant in pastoralists inhabited ASALs of Kenya that largely lack clean water and sanitation, and are increasingly being ravaged by prolonged severe droughts occasioned by climate change. The undernourished livestock in these areas will increasingly be more vulnerable to infections from environmental bacterial contaminants leading to the likelihood of escalated mis-and over-use of antimicrobials that will likely further aggravate AMR. Thus, our findings represent a significant contribution towards AMR mitigation. However, to attain field deployment of our designed vaccine several limitations of our study namely lack of molecular dynamics (MD) simulations data for the vaccine candidate in complex with TLR2 and wet-lab experiments will need to addressed. Besides, experimental assays in a mouse model to ascertain IgG titer need to be undertaken prior to bacterial challenge studies.

## Data Availability

The original contributions presented in the study are included in the article/**Supplementary Material**. Further inquiries can be directed to the corresponding author.
