# Supplementary material for: In silico design and testing of a multi-epitope camel mastitis vaccine candidate for development
Source: Front Cell Infect Microbiol. 2026 May 29;16:1753101. doi: 10.3389/fcimb.2026.1753101 (PMC13260313; doi:10.3389/fcimb.2026.1753101)
Supplement: Supplementary file 1 [file DataSheet1.docx]

Supplementary Table 1: Physicochemical properties of the top VaxiJen predicted antigenic *S. aureus* and *S. agalactiae* proteins. Theoretical pI, molecular weight, instability index, aliphatic index, GRAVY

| **Bacterial source** | **GenBank Accession** | **Protein Name** | **Length (amino acids)** | **Theoretical pI** | **Molecular Weight** | **Instability Index** | **Aliphatic Index** | **Grand average of hydropathicity (GRAVY)** |
| --- | --- | --- | --- | --- | --- | --- | --- | --- |
| *S. aureus* | MDF3296226.1 | Hypothetical protein P3G69_09200 | 30 | 8.21 | 3513.26 | 31.25 | 143 | 1.307 |
| *S. aureus* | MDF3345085.1 | Cell surface protein, partial | 284 | 4.25 | 28592.60 | 15,28 | 24.19 | -1.454 |
| *S. aureus* | MDF3296234.1 | Hypothetical protein P3G69_09240 | 66 | 9.10 | 7643.84 | 55.70 | 94.55 | -0.756 |
| *S. aureus* | MDF3296909.1 | DUF4887 domain-containing protein | 209 | 9.07 | 24070.16 | 64.82 | 34.16 | -1.974 |
| *S. aureus* | MDF3294667.1 | Elastin-binding protein EbpS | 486 | 5.92 | 53186.50 | 38.83 | 42.30 | -1.503 |
| *S. aureus* | MDF3294497.1 | Hypothetical protein P3G69_00220 | 39 | 9.69 | 4594.92 | 86.23 | 30.26 | -1.905 |
| *S. aureus* | MDF3295037.1 | Type 1 toxin-antitoxin system Fst family toxin | 35 | 9.78 | 4012.76 | 25.61 | 94.86 | 0.177 |
| *S. aureus* | MDF3296810.1 | Pathogenicity island protein | 48 | 4.53 | 5395.55 | 37.20 | 138.12 | 1.142 |
| *S. agalactiae* | MCP9190290.1 | Cell wall synthase accessory phosphoprotein MacP | 80 | 9.10 | 9396.97 | 59.85 | 113.37 | -0.285 |
| *S. aureus* | MDF3295131.1 | Hypothetical protein P3G69_03545 | 61 | 9.62 | 7611.01 | 44.62 | 59.02 | -0.546 |
| *S. aureus* | MDF3343883.1 | Hypothetical protein P3G69_08555 | 61 | 9.62 | 7578.95 | 45.57 | 63.77 | -0.508 |
| *S. aureus* | MDF3294606.1 | Hypothetical protein P3G69_00775 | 48 | 7.98 | 5776.79 | 30.94 | 105.62 | 0.108 |
| *S. aureus* | MDF3296979.1 | VraH family | 73 | 4.26 | 8761.12 | 32.18 | 94.79 | 0.103 |
| *S. aureus* | MDF3294509.1 | Preprotein translocase subunit YajC | 86 | 9.77 | 9671.39 | 31.26 | 107.56 | 0.102 |
| *S. aureus* | MDF3294841.1 | Hypothetical protein P3G69_02025 | 32 | 9.43 | 3831.64 | 37.24 | 82.19 | -0.487 |
| *S. aureus* | MDF3295053.1 | Putative metal homeostasis protein | 34 | 12.19 | 4069.92 | 86.24 | 89.12 | -1.229 |
| *S. aureus* | MDF3296041.1 | Hypothetical protein P3G69_08260 | 35 | 4.20 | 4041.80 | 17.03 | 136.29 | 1.491 |
| *S. agalactiae* | MCP9190874.1 | PDZ domain-containing protein | 342 | 9.71 | 37421.21 | 21.60 | 89.47 | -0.237 |
